# MaveMD: A functional data resource for genomic medicine

**DOI:** 10.1101/2025.11.15.25336228

**Published:** 2025-11-19

**Authors:** Abbye E. McEwen, Jeremy Stone, Malvika Tejura, Pankhuri Gupta, Benjamin J. Capodanno, Estelle Y. Da, Sally B. Grindstaff, Nick Moore, David Reinhart, Ashley E. Snyder, Andrew B. Stergachis, Lea M. Starita, Douglas M. Fowler, Alan F. Rubin

**Affiliations:** 1 Department of Laboratory Medicine and Pathology, University of Washington, Seattle, WA, USA; 2 Brotman Baty Institute for Precision Medicine, University of Washington, Seattle, WA, USA; 3 Department of Genome Sciences, University of Washington, Seattle, WA, USA; 4 Division of Medical Genetics, Department of Internal Medicine, University of Washington, Seattle, WA, USA; 5 Bioinformatics Division, Walter and Eliza Hall Institute of Medical Research, Parkville, VIC, Australia; 6 Department of Bioengineering, University of Washington, Seattle, WA, USA; 7 Department of Medical Biology, University of Melbourne, Parkville, VIC, Australia

## Abstract

Variant interpretation remains one of the most significant challenges in clinical genetics. Variants of uncertain significance (VUS) undermine precision medicine implementation because they have an unknown relationship to disease and cannot be used for clinical decision-making. While evidence from multiplexed assays of variant effect (MAVEs) and other functional assays can help classify variants, major barriers prevent routine use in clinical variant classification, including fragmentation across multiple repositories, insufficient data standards, and the need to calibrate assays clinically. Here we address these challenges by presenting a new interface for the MaveDB database called MaveMD (MAVEs for MeDicine) that integrates with external resources such as ClinVar and the ClinGen Allele Registry, displays clinical evidence calibrations, provides intuitive visualizations, and exports structured evidence compatible with ACMG/AMP variant classification guidelines. MaveMD implements automatic mapping of MaveDB datasets to the human reference genome, dramatically simplifying the clinical translation of new MAVE data. We also defined a new metadata model after curating 438,318 variant effect measurements from 74 MAVE datasets spanning 32 disease-associated genes, and created an interface aimed at enabling effective clinical decision-making. Thus, MaveMD makes MAVE data accessible and easily usable for variant classification, and will scale seamlessly with future data generation efforts to empower the use of MAVE evidence in clinical practice.

## Introduction

Clinical interpretation of genetic variants is challenging, often because of variants of uncertain significance (VUS) that cannot be used for diagnosis or to guide clinical decision-making. The widely-used American College of Medical Genetics and Genomics (ACMG) and Association for Molecular Pathology (AMP) variant classification framework integrates evidence including population frequency, familial segregation, case-control studies, computational predictions, and functional assays to classify variants as pathogenic, likely pathogenic, VUS, likely benign, or benign (Richards et al., 2015). Each piece of evidence can support pathogenicity or benignity and is weighted as very strong, strong, moderate, supporting, or stand-alone. The final variant classification is determined by combining evidence using a point-based system (Richards et al., 2015; Tavtigian et al., 2018). If the point total does not meet the threshold for likely pathogenic or likely benign, the variant is classified as VUS. Up to 40% of individuals undergoing genetic testing receive VUS results (Chen et al., 2023; Chrysafi et al., 2023; Rehm et al., 2023), disproportionately affecting individuals of non-European ancestry (Martin et al., 2024; Ndugga-Kabuye and Issaka, 2019).

Data from multiplexed assays of variant effect (MAVEs) are a powerful source of evidence for classifying genetic variants and resolving VUS (McEwen et al., 2025). These high-throughput experiments simultaneously characterize thousands of variants in a single assay. Each variant receives a functional score from the assay, generating a comprehensive variant effect map for the target gene (Findlay, 2021; Starita et al., 2017; Tabet et al., 2022; Weile and Roth, 2018). Incorporating MAVE data into variant classification workflows allows reclassification of ~55% of VUS across well-studied genes (Fayer et al., 2021; McEwen et al., 2025), and can play a major role in reducing classification disparities for individuals from non-European ancestries (Dawood et al., 2024). Although MAVE-derived evidence is valuable for clinical variant classification, its use has been constrained because of barriers to discovering and accessing MAVE data, as well as the lack of necessary expertise amongst clinicians to evaluate data quality, assay utility, and evidence strength (Allen et al., 2025; Park et al., 2025; Villani et al., 2025).

MAVE data has been difficult to access because it is fragmented across supplemental tables of publications, websites maintained by individual laboratories, gene-specific databases, general repositories like GitHub and Zenodo, and the MaveDB community database. This dispersion creates an untenable burden for clinical laboratories compounded by data loss as supplemental tables, websites and databases become unavailable over time (Briney, 2024; Costa et al., 2023; Federer, 2022; Lemaire et al., 2025; Mighton et al., 2022). Even if clinical users can locate MAVE data, they need key metadata to evaluate its clinical utility and inform its use, including information about the model system and experiment design, the molecular phenotype assessed, and the types of variants the assay detects (e.g., splicing, dominant negative, loss of function, or gain of function). Additionally, clinicians need to calibrate the data, a process that transforms MAVE-derived functional scores into evidence for clinical variant classification. Calibration relies on clinical control variants with previously-established pathogenic or benign classifications from sources like ClinVar (Landrum et al., 2025) to serve as benchmarks for determining evidence strength (Brnich et al., 2019). Many MAVE datasets have been calibrated, but these evidence assignments are often also buried in supplementary tables and therefore are not easily discoverable or usable. Moreover, multiple calibration methods are available, including methods that assign variant-specific evidence strength (van Loggerenberg et al., 2023; Zeiberg et al., 2025) and the ClinGen-specified OddsPath method (Brnich et al., 2019), a useful quantitative metric that reflects an assay’s ability to distinguish between benign and pathogenic variants.

**Figure 1:**
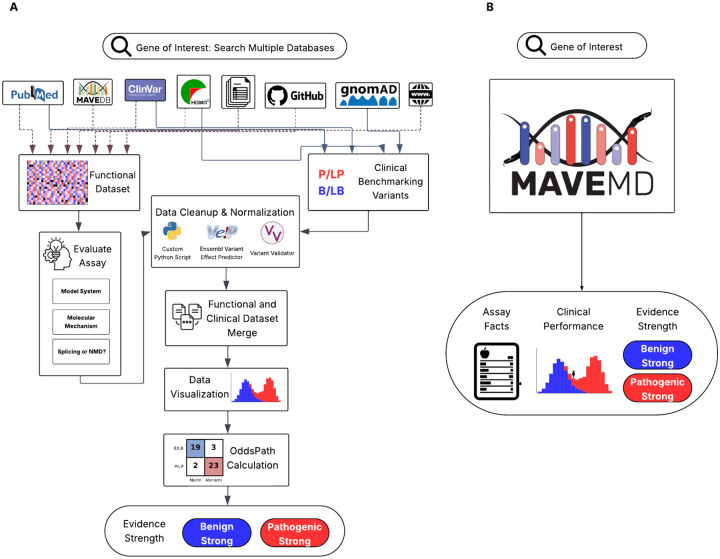
Comparison of workflows for using MAVE data in a clinical context. A) Currently clinical users must retrieve data from multiple databases, perform data cleanup and normalization, merge MAVE datasets with clinical control variants, assess clinical performance, and calibrate the functional/MAVE data to determine evidence strength. (B) The streamlined MaveMD interface displays the results of those steps in a single platform, providing direct access to key assay metadata, visualization of clinical performance, and evidence strength classifications in an accessible, clinician-friendly format.

To promote data discoverability, MaveDB has been established as the community database for MAVE data with 1,995 total datasets, including variant effect measurements in 455 human genes, 255 of which have a known relationship to human disease. In addition to variant functional scores and basic information about the assay target, MaveDB stores text metadata designed to meet the needs of data scientists and researchers, including a short description of the assay, an abstract, and abbreviated methods (Esposito et al., 2019; Rubin et al., 2025). However, MaveDB lacks features needed by clinical users, including individual variant search, standardized human genomic coordinate mapping, clinically-oriented metadata, and clinical evidence strength for individual variants. Thus, even if clinical users downloaded a dataset from MaveDB, they would then need to merge information from diverse resources before using the MAVE data (Figure 1A).

The clinical utility of MAVE data depends on understanding the relationship between the specific assay molecular phenotype (*i.e*., what the assay is measuring) and the gene-disease relationship being evaluated. An assay must measure a molecular function directly relevant to the disease pathophysiology to provide meaningful evidence. Therefore, clinicians must determine the molecular phenotype measured, the variant types the assay reliably detects (*e.g*., loss-of-function, gain-of-function, and dominant negative), and if an assay can detect splicing variants or variants predicted to undergo nonsense mediated decay (NMD). Without this key information, even well-calibrated MAVE data may lead to incorrect variant classifications if applied to inappropriate gene-disease contexts, underscoring the importance of displaying easily understandable assay metadata. Furthermore, MAVE datasets have grown in scale, with some assessing more than 10,000 variant effects (Radford et al., 2023), and newer calibration methods are sufficiently complex (Zeiberg et al., 2025) that a centralized repository is needed to provide calibrated data to clinical laboratories.

To bridge this gap, we present MaveMD (MAVEs for MeDicine), a resource built on MaveDB and informed by surveys of clinical users to help clinicians discover, evaluate and use MAVE data (Figure 1B). MaveMD includes a new variant search function powered by ClinGen Allele IDs to ensure accuracy and portability between information systems (Pawliczek et al., 2018). We also developed and implemented a standardized, clinically-relevant metadata model based on existing minimum information standards (Claussnitzer et al., 2024), and adopted modern Global Alliance for Genomics and Health (GA4GH) standards to power sophisticated API-based data integration (Rehm et al., 2021; Wagner et al., 2021). MaveMD links MAVE functional scores to known pathogenic and benign clinical control variants from ClinVar, and exposes evidence strength assignments from two calibration methods (Brnich et al., 2019; Zeiberg et al., 2025). The clinical interface is accessible via REST API for programmatic access or the web front-end with standardized and intuitive visualizations allowing users to evaluate assay results, explore key metadata, and examine calibrations. We populated MaveMD with an initial set of 74 curated and calibrated MAVE datasets with 438,318 distinct variant effect measurements and 268,451 evidence strength assignments for 32 clinically-relevant genes, including 10 actionable genes on the ACMG secondary findings list (Lee et al., 2025). Lastly, MaveMD is integrated with the Impact of Genetic Variation on Function (IGVF) Consortium’s Catalog, providing access to MAVE data as it is generated (IGVF Consortium, 2024). Thus, MaveMD builds upon MaveDB by adding extensive metadata curation, evidence calibration, and visualizations designed to support routine integration of functional evidence into variant classification workflows.

## Methods

### Data curation and selection criteria

To establish a comprehensive collection of clinically relevant MAVE datasets, we performed systematic curation of published functional studies. We identified candidate datasets by searching MaveDB, querying ClinVar for publications citing foundational MAVE methodology or clinical assay calibration papers (Brnich et al., 2019; Fayer et al., 2021; Fowler and Fields, 2014), and soliciting expert recommendations from the genetics community. To ensure focus on genes with sufficient evidence for clinical interpretation, we only included genes with moderate or greater disease associations as determined by either the ClinGen Gene-Disease Validity Working Group classifications or the GenCC database (DiStefano et al., 2022).

A multidisciplinary curation team led by a molecular genetic pathologist (A.E.M.) and including a senior graduate student (M.T.) and genetic counselor (P.G.) met weekly to review curations, resolve ambiguities, and ensure consistency. Systematic metadata was extracted across six key domains encompassing over 180 individual fields. Dataset identification and availability metadata included gene identifiers (HGNC symbols and IDs), publication details (PMID, year, first author), data locations (MaveDB URNs, supplemental tables, repositories), and accessibility status. Assay design parameters captured experimental methodology (arrayed vs. pooled, saturation vs. targeted variants), model system specifications (organism, cell line or yeast details), library strategies (variant types, mutagenesis approach, delivery method), phenotypes measured, and molecular processes investigated. Technical performance metadata included replicate structure, variance analysis between replicates, and comparisons with other functional assays. Functional scoring included methods used for calculating variant effect scores and associated statistics. Score classification described methods for assigning functional classifications and assay thresholds for each functional class. Clinical performance metrics captured the use and source of pathogenic and benign clinical control variants, evidence strength assignments per ACMG/AMP guidelines (OddsPath), and performance statistics (sensitivity, specificity, PPV, ROC-AUC). This comprehensive metadata collection ensured standardized data capture across all curated datasets and directly informed the design requirements for MaveMD.

### Data wrangling and standardization

The heterogeneous nature of published MAVE data necessitated extensive standardization before datasets could be submitted to MaveDB, which underlies MaveMD. Source data, including variant functional scores, were collected in diverse supplemental table formats (Excel, CSV, TSV, PDF) with inconsistent column naming conventions, variant nomenclature, and score representations. We developed a systematic pipeline to transform this information into MaveDB-compatible formats.

For each curated dataset, we performed manual inspection to identify the data’s structure, including the variant representation format (amino acid substitutions, nucleotide changes, mixed nomenclature, custom notation), score columns and their meanings (raw scores, normalized scores, confidence intervals, standard errors), replicate structure when present, and any additional metadata columns requiring preservation. Converting variant descriptions to the HGVS-based format (den Dunnen et al., 2016) used by MaveDB was the most complex part of the process. Source datasets employed various nomenclature systems: single-letter amino acid codes, three-letter amino acid codes, nucleotide positions without reference sequences, genomic coordinates in multiple genome builds, and laboratory-specific custom notations. Following standardization and deposition into MaveDB, all datasets were mapped to the human reference genome using sequence alignment as previously described (Arbesfeld et al., 2025).

### Data counting and summarization

Datasets in MaveDB and elsewhere are counted as unique combinations of a functional assay and assay target. In MaveDB, this is tabulated at the level of the experiment record. For cases where the same raw sequencing data was analyzed in multiple ways to produce different sets of variant effect measurements, these were not considered unique datasets.

After data wrangling and standardization, the 74 curated datasets encompassed 438,318 variant effect measurements. This count reflects all assay-level measurements, where unique genetic variants tested in multiple assays contribute multiple counts to the total. After collapsing variants measured in multiple assays, the dataset contained 209,346 unique variants. In this manuscript, counts reported at the unique variant level are based on this collapsed dataset, while counts at the variant effect measurement level are taken from the complete set of assay-level measurements.

### Interface prototype development and assessment

To inform the development of the MaveMD interface, we utilized findings from a comprehensive needs assessment survey (Park et al., 2025). The survey captured responses from 190 genetics professionals between February and June 2024, who evaluated a prototype MAVE data dashboard featuring ~4,000 *BRCA1* variants. Participants evaluated eight specific interface features using Likert-scale questions, assessing each component’s potential utility for clinical variant interpretation. The dashboard included a histogram displaying MAVE functional score distributions colored by ClinVar significance with functional thresholds, OddsPath calculations, ACMG/AMP evidence codes, variant identifiers, functional consequences, scores, and associated errors.

### Software implementation

MaveMD is built on the MaveDB open source codebase and shares many key components (Rubin et al., 2025). Briefly, the software is implemented in Python and JavaScript using FastAPI and Vue.js, respectively, and a Postgres database. MaveDB and MaveMD source code is available under the AGPL-3.0 license (see Data and code availability). They are deployed on Amazon Web Services as a series of Docker containers and other services (Supplemental Figure S1).

## Results

### Understanding MaveMD requirements

Since MaveDB’s launch, clinical users have consistently requested features such as variant search that would help them leverage MAVE data in their practice. We surveyed 190 respondents, predominantly laboratory medical geneticists (23%) and variant review scientists (23%), using a mockup MaveMD interface with different proposed features: metadata about how the assay was performed, functional classification thresholds (e.g., loss-of-function or functionally normal), standardized variant identifiers, a visualization comparing pathogenic and benign control variants to assay results, and calibrated evidence strength using ACMG/AMP v3 evidence codes (Park et al., 2025; Richards et al., 2015).

Response to the mockup was overwhelmingly positive, with strong support for all the proposed features (Park et al., 2025). However, while 94% of respondents preferred having access to standardized ACMG evidence codes (PS3/BS3), only 68% stated they would directly use these codes if displayed. This gap revealed that clinical users prefer transparency and want to review both the evidence codes and the underlying supporting data before making decisions. These findings translated into clear requirements for MaveMD: robust mapping of MAVE variants to the reference genome, variant search functionality, inclusion of metadata essential for clinical interpretation, and presentation of calibrations with underlying clinical control variants.

### Contextualizing MAVE data for clinical application

Inspired by the results of our survey, we systematically curated 74 clinically relevant MAVE datasets across 32 disease-associated genes (Adamovich et al., 2022; Boettcher et al., 2019; Bolognesi et al., 2019; Buckley et al., 2024; Fayer et al., 2021; Findlay et al., 2018; Fortuno et al., 2021; Gebbia et al., 2024; Gersing et al., 2024, 2023; Giacomelli et al., 2018; Gilbert et al., 2024; Glazer et al., 2020; Grønbæk-Thygesen et al., 2024; Hu et al., 2024; Jia et al., 2021; Jiang et al., 2022; Kato et al., 2003; Kozek et al., 2020; Li et al., 2023; Lo et al., 2023; J. G. Ma et al., 2024; K. Ma et al., 2024; Matreyek et al., 2018; McDonnell et al., 2024; Meitlis et al., 2020; Mighell et al., 2018; Muhammad et al., 2024; Olvera-León et al., 2024; O’Neill et al., 2024; Popp et al., 2025; Radford et al., 2023; Sahu et al., 2025, 2023; Shepherdson et al., 2024; Sun et al., 2020; Sung et al., 2024; van Loggerenberg et al., 2023; Wan et al., 2019; Waters et al., 2024; Weile et al., 2017; Zheng et al., 2022). Our curation data model included all metadata outlined in the MAVE minimum information standards (Claussnitzer et al., 2024) augmented with additional fields critical for clinical use, such as the molecular mechanism measured by the assay, variant consequences detected by the assay, and whether the assay could detect splicing variants or variants that would be subject to NMD in a physiologic context. Ultimately, each dataset underwent comprehensive metadata curation across six domains: dataset identification, assay design parameters, technical performance metrics, functional classifications, score calculations, and clinical performance statistics.

To understand the coverage of our MAVE data, we analyzed the overlap between variants characterized in our curated datasets and existing clinical resources. Of the 209,346 unique total variants across all curated MAVEs, 13.49% (28,246) were present in ClinVar, with 4.78% (10,017) having confident clinical classifications (pathogenic/likely pathogenic or benign/likely benign). Notably, 26.37% (55,206) of MAVE-characterized variants were absent from both ClinVar and gnomAD, representing potentially rare variants not previously observed in population or clinical cohorts.

**Figure 2:**
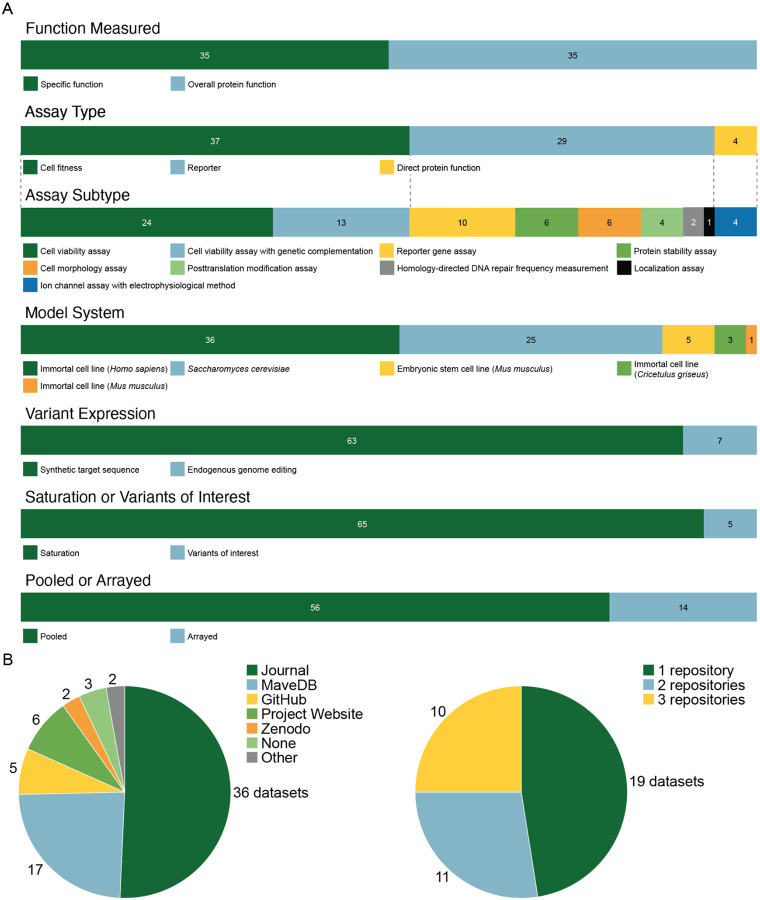
Summary of curated clinically-relevant MAVE datasets. A) The stacked bar plots show the proportion of datasets split across several metadata categories. The legend for each bar is shown below. B) The pie chart demonstrates the location of MAVE dataset deposition at the time of publication.

Curated datasets reflected diverse experimental approaches, contributing to the difficulties clinicians face when trying to evaluate assays. Arguably the most essential challenge is understanding the type of assay being performed, and this diversity emphasizes the importance of organizing and displaying interpretable metadata (Figure 2A). Of the 74 datasets we curated, six combined multiple dissimilar functional assays and were excluded. We found that assays were evenly split between those that measured overall protein function and those that measured a specific function. Assay types included cell fitness (49%), reporters (38%), and direct protein function (5%), with assay types further split into subtypes to help guide application of each dataset.

The assay systems also differed substantially across our curated datasets, with most experiments performed in various mammalian cell lines (64%) and the remainder in yeast cells. Variants were mostly generated using cDNA-based approaches that required introduction of a synthetic target sequence (86%), with the remaining minority using endogenous genome editing techniques such as saturation genome editing. The overwhelming majority (93%) of datasets used saturation mutagenesis, measuring the effects of all possible variants across a region or gene. Of the assays we curated, most were pooled assays, as expected for deep mutational scanning and other MAVE methods (80%), but a few used arrayed formats for technical or historic reasons.

As part of curation, we ensured that each dataset was uploaded to MaveDB. Of the 42 publications analyzed, 17 (41%) already had data deposited, 17 (41%) only had data in supplemental tables on journal websites, and one (2.4%) could only be found on a project-specific academic website, highlighting the discoverability challenges facing clinical users (Figure 2B). One curated dataset was associated with links that were no longer functional but available through other sources, and one dataset could not be curated because the only data source was a non-functional website. Likewise, two datasets were excluded because they were in PDF files that could not be easily converted. In total, we curated key functional assay data and clinically-oriented metadata for 209,346 variants across 32 genes totalling 438,318 unique variants effect measurements.

For MAVE data to be translated into functional evidence, clinical control variants must be compared to functional classifications derived from variant effect measurements. While most datasets provided a functional classification for each variant or included information such as clearly defined score intervals (58%), the remaining studies did not. Additionally, only a minority of datasets included clinical evidence strength calculations (27%) with 16 using the OddsPath method and four using a variant-specific log-likelihood ratio method.

### MaveMD clinical interface implementation

**Figure 3:**
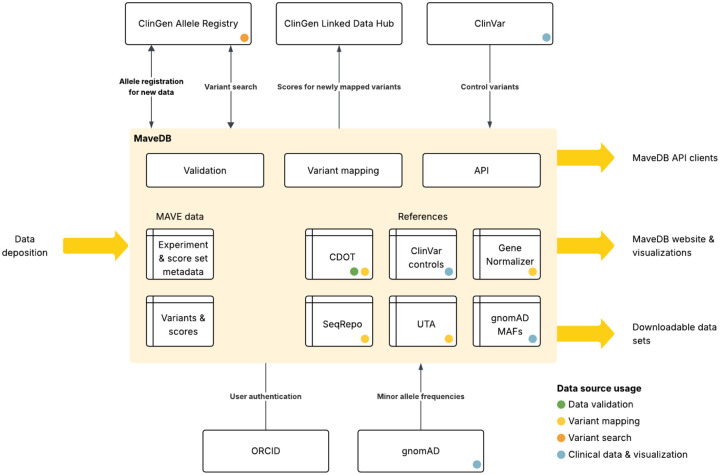
Data flow for clinically-relevant datasets in MaveDB. This schematic shows the various data sources and services used by MaveDB and MaveMD as part of the mapping and annotation process. Sources of reference information include the cdot transcript resolver (https://github.com/SACGF/cdot), classified variants from ClinVar, Gene Normalizer for resolving ambiguous sequences (https://github.com/cancervariants/gene-normalization), SeqRepo for storing a local collection of sequences (Hart and Prlić, 2020), the Universal Transcript Archive (UTA) for storing aligned transcripts (https://github.com/biocommons/uta/), and population minor allele frequencies from gnomAD (Chen et al., 2024).

In developing the MaveMD interface, we were guided by three key priorities: variant search functionality, representation of metadata essential for clinical interpretation, and presentation of calibrations with underlying clinical control variants (Park et al., 2025). Variant search presented significant challenges due to the diversity of transcripts, variant identifiers, variant formats, and coordinate systems. To address this complexity, we utilized the ClinGen Allele Registry, which provides and maintains universal identifiers for genetic variants (Pawliczek et al., 2018). We first implemented an automatic mapping process that determines the corresponding human genomic variant for every variant in MaveDB that comes from a human sequence, regardless of the assay system (Arbesfeld et al., 2025), allowing us to create or retrieve associated ClinGen Allele IDs from the Registry, as well as display annotations from other resources such as gnomAD (Figure 3).

Having mapped variants and obtained ClinGen Allele IDs, we were able to implement two types of search designed to support commonly-used variant nomenclatures from clinical reporting systems. Users can search for variants with standard HGVS strings or through a structured “fuzzy search” interface that requires a gene symbol, variant type (protein or cDNA), position, and reference and alternate alleles (Supplemental Figure S2). Search results display available MAVE datasets and provide direct access to a purpose-built clinical dashboard, including the option to choose between different functional datasets when multiple measurements are available (Supplemental Figure S3). The top panel displays variant identifiers and external links, including ClinGen Allele ID and ClinVar Variant ID, enabling integration and exploration in other clinical resources. Users are also able to link directly to a MaveMD variant from the corresponding variant page in the ClinGen Allele Registry via the ClinGen Linked Data Hub. The variant’s functional consequence is also prominently displayed with a color-coded functional classification.

Upon searching for a specific variant, MaveMD provides a clinical view designed to help users access and interpret MAVE data and clinical metadata for variant classification. We developed a standardized presentation format that transforms MAVE data into actionable information, reducing the workload for clinical users while maintaining scientific rigor and transparency about assay capabilities and limitations. The interface contains three major features: assay fact labels, interactive visualizations displaying functional scores, and evidence calibration information.

**Figure 4:**
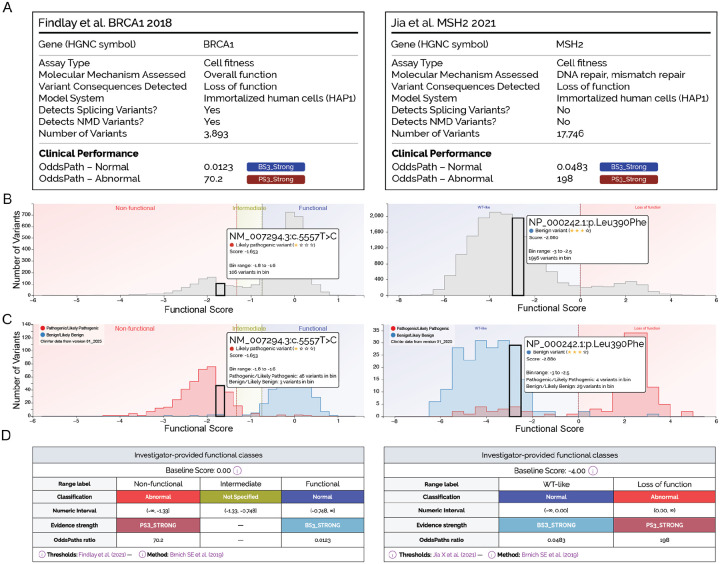
MaveMD data display elements. The MaveMD interface features multiple different visualizations and visual summaries. A) Each dataset has its own assay facts box that summarizes the key details that will help a user evaluate its clinical utility. Where needed, additional references are denoted with a superscript number that links to the full reference on the associated MaveDB or MaveMD page. B) Interactive histograms show the full distribution of variant scores in an assay and the relative position of a selected variant. Vertical bars also denote any evidence- or function-based cutoffs. C) Another interactive histogram displays the distribution of variant scores for variants from ClinVar. This view is intended to help users evaluate the clinical relevance of an assay based on the separation between independently-classified pathogenic and benign variants. Users can choose different snapshots of the ClinVar database. D) OddsPath and the associated evidence codes and score ranges are shown in a table below the histogram.

The first major feature, assay fact labels, addresses the need for rapid assessment of a functional assay’s relevance to a given clinical application (Figure 4A). They display key characteristics including assay type (cell fitness, reporter, direct protein function) and molecular phenotype assessed, model system (*e.g*., human cells or yeast), variant functional consequences detected (loss of function, gain of function, dominant negative), the ability to detect splicing or NMD variants, and the total number of variants tested. A clinical performance section in each assay fact label provides at-a-glance evidence strength assessments using color-coded ACMG/AMP-style evidence codes and OddsPath values for both normal and abnormal function or a note if OddsPath values are not available. Thus, our assay fact labels highlight critical information for clinicians, allowing rapid comparison between different MAVE datasets using a consistent format, and reveal assays meeting specific clinical needs.

Figure 4A presents two examples: the left panel illustrates *BRCA1* saturation genome editing results that measures overall BRCA1 function related to cell survival and detects both splicing and NMD variants (Findlay et al., 2018). The right panel shows a cell survival based assay of DNA mismatch repair for MSH2, which, due to its cDNA-based approach, cannot detect variants affecting splicing or causing NMD (Jia et al., 2021). This distinction is clinically relevant since a laboratory investigating a putative splice variant needs to quickly identify that cDNA-based experimental methods might yield falsely normal results.

The second major feature facilitates the assessment of assay performance via an interactive histogram displaying the distribution of functional scores across all tested variants. This visualization highlights user-selected variants within the broader context of all variant effect measurements in the assay. In the “Overall Distribution” view, the histogram features clear demarcation of author-specified functional classes with colored backgrounds (for example, blue for “wild type-like” or “normal activity”, red for “loss of function” or “abnormal activity”, yellow for “intermediate”) (Figure 4B). This color coding is especially helpful for a dataset like MSH2, as in contrast to most MAVE readouts, lower functional scores actually indicate normal protein activity.

The third major feature provides transparent access to evidence calibrations. By toggling to a “Clinical Controls View”, the interactive histogram displays only the functional scores for classified ClinVar variants and shows OddsPath calculations and evidence strength assignments (Figure 4C and D). Variants are color-coded by their ClinVar status, with red for pathogenic/likely pathogenic and blue for benign/likely benign, alongside statistics showing the number of clinically classified variants in each functional score bin. This visualization immediately reveals whether functional scores are concordant with previously-classified clinical variants and highlights potential discordances requiring further investigation. Recognizing that multiple calibration algorithms already exist and continue to be developed, MaveMD allows users to toggle between the results of different methods. For example, we currently provide calibrations from the OddsPath and variant specific Zeiberg et al. methods (Supplemental Figure S4) (Brnich et al., 2019; Zeiberg et al., 2025).

Users can also download the MAVE datasets, calibrations, and metadata they need to make their own assessments. To preserve the rich contextual metadata required for interpretation, we use the GA4GH Variant Representation Specification (VRS) for describing genomic variants (Wagner et al., 2021) and the GA4GH Variant Annotation Specification (VA-Spec) for describing assay scores, functional annotations, and clinical evidence strength (https://github.com/ga4gh/va-spec).

## Discussion

Our systematic curation and centralization of MAVE datasets and the new MaveMD interface directly confronts the data dispersion crisis plaguing clinical genetics. The fragmentation we documented, with datasets scattered across supplemental tables, institutional websites, and various repositories, creates a substantial burden for clinical laboratories already managing heavy caseloads. Prior to this work, clinical laboratories faced the daunting task of independently evaluating each functional assay, requiring both deep domain expertise and computational skills often unavailable in clinical settings. By establishing MaveMD as a central hub with persistent identifiers and standardized formats, we eliminate the need for exhaustive multi-platform searches while enabling long-term data discovery and preservation.

Fundamental challenges remain that both limit the current implementation and point toward future solutions. The inconsistent representation of measurement uncertainty across datasets complicates the incorporation of variant-level confidence into clinical interpretation. Much of this heterogeneity is driven by the diverse landscape of scoring methods available to researchers (Çubuk et al., 2025), as well as the distinct sources of error present in different experimental designs. Assembling a harmonized dataset as we have done here is the first step in tackling this systematically by further improving metadata annotations and identifying candidates for large-scale reanalysis.

The most complex unresolved issue is dealing with conflicting MAVE data. When multiple assays for the same variant yield discordant results, clinical users lack a clear framework for reconciliation. Because assays may measure different molecular properties, a system like ClinVar’s transparent display of conflicting interpretations is not suitable. A variant could show normal protein abundance in one MAVE and demonstrate impaired enzymatic activity in another; both results may be correct, but their integration requires sophisticated understanding of disease mechanisms and the relative importance of different molecular functions.

Our current approach of displaying all available MAVE data with associated metadata promotes transparency, but leaves the task of reconciliation to clinical users, and the required expertise may exceed what is reasonable to expect from clinical laboratories. Datasets assembled in MaveMD can be used to train machine learning models generating composite functional scores that leverage the complementary strengths of different experimental approaches, as has been demonstrated for TP53 (Calhoun et al., 2025; Fayer et al., 2021). MaveDB already supports multiple score calculations for a single assay, as well as model-based combinations of multiple assays in its data model. While large-scale reanalysis and model training is out of scope for MaveDB’s current role as a data sharing platform, disseminating such results through MaveMD would be straightforward.

While MaveMD significantly improves access to MAVEs for clinical use, important limitations remain in connecting assays to specific gene-disease relationships. Currently, we provide comprehensive assay metadata including molecular phenotypes measured and variant types detected, but clinical users must determine whether a given assay is appropriate. Furthermore, our current implementation displays all pathogenic and benign clinical controls from ClinVar regardless of disease association, as disease annotations are often incomplete, inconsistent, or unreliable. This approach may include variants related to diseases with different molecular mechanisms than the condition of interest, potentially affecting calibration accuracy. Future development should prioritize integration with a centralized, well-curated resource that systematically maps gene-disease relationships to their underlying molecular mechanisms. Such a resource would enable MaveMD to automatically flag which assays are most relevant for specific clinical indications. Until this information is standardized and computationally accessible, clinical users must exercise careful judgment in selecting and applying MAVE evidence, considering both the molecular basis of the disease in question and the specific functional readout of each assay. Our curation experience illustrates the intensive effort required to extract, standardize, and calibrate each dataset, and scaling this manual process to accommodate the accelerating pace of MAVE publications is a fundamental challenge. By reporting crucial insights into what information is essential for clinical interpretation, our expanded data model will help shift this work from post-publication curation to data deposition, while also streamlining the process for data submitters by moving away from free text towards more intuitive structured metadata.

MaveMD and our associated expert curations demonstrate that the primary barriers to implementing functional evidence in the clinic are logistical rather than scientific. By addressing data centralization, metadata and format standardization, and calibration complexity, we have created a foundation for routine use of MAVE and other functional data that we hope will satisfy clinical needs now and into the future. However, challenges around measurement uncertainty, conflicting results, and sustainable maintenance require continued community effort. The long-term success of functional evidence in clinical practice depends on establishing sustainable infrastructure for data generation, data sharing, and platform maintenance. As functional assays become increasingly sophisticated and widespread, parallel advances in computational infrastructure, experimental and analytical methods, and community standards will be essential to realize their full potential for resolving variants of uncertain significance and reducing disparities in genetic medicine.

## Supplementary Material

1

## Data Availability

MaveDB, MaveMD, and associated online documentation are available at https://mavedb.org. Source code for MaveDB and MaveMD is available at https://github.com/VariantEffect/mavedb-api (for the back-end application) and https://github.com/VariantEffect/mavedb-ui (for the website). The present paper describes version 2025.5.0.

## References

[R1] Adamovich A.I., Diabate M., Banerjee T., Nagy G., Smith N., Duncan K., Mendoza Mendoza E., Prida G., Freitas M.A., Starita L.M., Parvin J.D., 2022. The functional impact of BRCA1 BRCT domain variants using multiplexed DNA double-strand break repair assays. Am J Hum Genet 109, 618–630. 10.1016/j.ajhg.2022.01.019 35196514 PMC9069074

[R2] Allen S., Garrett A., Rowlands C.F., Durkie M., Burghel G.J., Robinson R., Callaway A., Field J., Frugtniet B., Palmer-Smith S., Grant J., Pagan J., McDevitt T., Snape K., Hanson H., McVeigh T., Adams D.J., Findlay G.M., Villani R.M., Spurdle A.B., Turnbull C., CanVIG-UK Consortium, 2025. Validating data from multiplex assays of variant effect: A CanVIG-UK national survey of NHS clinical scientists. Am J Hum Genet 112, 1479–1488. 10.1016/j.ajhg.2025.04.006 40480200 PMC12256791

[R3] Arbesfeld J.A., Da E.Y., Stevenson J.S., Kuzma K., Paul A., Farris T., Capodanno B.J., Grindstaff S.B., Riehle K., Saraiva-Agostinho N., Safer J.F., Casper J., Haeussler M., Milosavljevic A., Foreman J., Firth H.V., Hunt S.E., Iqbal S., Cline M.S., Rubin A.F., Wagner A.H., 2025. Mapping MAVE data for use in human genomics applications. Genome Biol 26, 179. 10.1186/s13059-025-03647-x 40563119 PMC12188674

[R4] Boettcher S., Miller P.G., Sharma R., McConkey M., Leventhal M., Krivtsov A.V., Giacomelli A.O., Wong W., Kim J., Chao S., Kurppa K.J., Yang X., Milenkowic K., Piccioni F., Root D.E., Rücker F.G., Flamand Y., Neuberg D., Lindsley R.C., Jänne P.A., Hahn W.C., Jacks T., Döhner H., Armstrong S.A., Ebert B.L., 2019. A dominant-negative effect drives selection of TP53 missense mutations in myeloid malignancies. Science 365, 599–604. 10.1126/science.aax3649 31395785 PMC7327437

[R5] Bolognesi B., Faure A.J., Seuma M., Schmiedel J.M., Tartaglia G.G., Lehner B., 2019. The mutational landscape of a prion-like domain. Nat Commun 10, 4162. 10.1038/s41467-019-12101-z 31519910 PMC6744496

[R6] Briney K.A., 2024. Measuring data rot: An analysis of the continued availability of shared data from a Single University. PLoS One 19, e0304781. 10.1371/journal.pone.0304781 38838010 PMC11152257

[R7] Brnich S.E., Abou Tayoun A.N., Couch F.J., Cutting G.R., Greenblatt M.S., Heinen C.D., Kanavy D.M., Luo X., McNulty S.M., Starita L.M., Tavtigian S.V., Wright M.W., Harrison S.M., Biesecker L.G., Berg J.S., Abou Tayoun A.N., Berg J.S., Biesecker L.G., Brenner S.E., Cutting G.R., Ellard S., Greenblatt M.S., Harrison S.M., Karbassi I., Karchin R., Mester J.L., O’Donnell-Luria A., Pesaran T., Plon S.E., Rehm H., Tavtigian S.V., Topper S., On behalf of the Clinical Genome Resource Sequence Variant Interpretation Working Group, 2019. Recommendations for application of the functional evidence PS3/BS3 criterion using the ACMG/AMP sequence variant interpretation framework. Genome Medicine 12, 3. 10.1186/s13073-019-0690-2 31892348 PMC6938631

[R8] Buckley M., Terwagne C., Ganner A., Cubitt L., Brewer R., Kim D.-K., Kajba C.M., Forrester N., Dace P., De Jonghe J., Shepherd S.T.C., Sawyer C., McEwen M., Diederichs S., Neumann-Haefelin E., Turajlic S., Ivakine E.A., Findlay G.M., 2024. Saturation genome editing maps the functional spectrum of pathogenic VHL alleles. Nat Genet 56, 1446–1455. 10.1038/s41588-024-01800-z 38969834 PMC11250436

[R9] Calhoun J.D., Dawood M., Rowlands C.F., Fayer S., Radford E.J., McEwen A.E., Turnbull C., Spurdle A.B., Starita L.M., Jagannathan S., 2025. Combining multiplexed functional data to improve variant classification. ArXiv arXiv:2503.18810v1. 10.1186/s13073-026-01715-w PMC13644118 42432739

[R10] Chen E., Facio F.M., Aradhya K.W., Rojahn S., Hatchell K.E., Aguilar S., Ouyang K., Saitta S., Hanson-Kwan A.K., Capurro N.N., Takamine E., Jamuar S.S., McKnight D., Johnson B., Aradhya S., 2023. Rates and Classification of Variants of Uncertain Significance in Hereditary Disease Genetic Testing. JAMA Netw Open 6, e2339571. 10.1001/jamanetworkopen.2023.39571 37878314 PMC10600581

[R11] Chen S., Francioli L.C., Goodrich J.K., Collins R.L., Kanai M., Wang Q., Alföldi J., Watts N.A., Vittal C., Gauthier L.D., Poterba T., Wilson M.W., Tarasova Y., Phu W., Grant R., Yohannes M.T., Koenig Z., Farjoun Y., Banks E., Donnelly S., Gabriel S., Gupta N., Ferriera S., Tolonen C., Novod S., Bergelson L., Roazen D., Ruano-Rubio V., Covarrubias M., Llanwarne C., Petrillo N., Wade G., Jeandet T., Munshi R., Tibbetts K., O’Donnell-Luria A., Solomonson M., Seed C., Martin A.R., Talkowski M.E., Rehm H.L., Daly M.J., Tiao G., Neale B.M., MacArthur D.G., Karczewski K.J., 2024. A genomic mutational constraint map using variation in 76,156 human genomes. Nature 625, 92–100. 10.1038/s41586-023-06045-0 38057664 PMC11629659

[R12] Chrysafi P., Jani C.T., Lotz M., Al Omari O., Singh H., Stafford K., Agarwal L., Rupal A., Dar A.Q., Dangelo A., Lam P., 2023. Prevalence of Variants of Uncertain Significance in Patients Undergoing Genetic Testing for Hereditary Breast and Ovarian Cancer and Lynch Syndrome. Cancers (Basel) 15, 5762. 10.3390/cancers15245762 38136308 PMC10742236

[R13] Claussnitzer M., Parikh V.N., Wagner A.H., Arbesfeld J.A., Bult C.J., Firth H.V., Muffley L.A., Nguyen Ba A.N., Riehle K., Roth F.P., Tabet D., Bolognesi B., Glazer A.M., Rubin A.F., 2024. Minimum information and guidelines for reporting a multiplexed assay of variant effect. Genome Biol 25, 100. 10.1186/s13059-024-03223-9 38641812 PMC11027375

[R14] Costa M., García S A., Pastor O., 2023. The consequences of data dispersion in genomics: a comparative analysis of data sources for precision medicine. BMC Med Inform Decis Mak 23, 256. 10.1186/s12911-023-02342-w 37946154 PMC10636939

[R15] Dawood M., Fayer S., Pendyala S., Post M., Kalra D., Patterson K., Venner E., Muffley L.A., Fowler D.M., Rubin A.F., Posey J.E., Plon S.E., Lupski J.R., Gibbs R.A., Starita L.M., Robles-Espinoza C.D., Coyote-Maestas W., Gallego Romero I., 2024. Using multiplexed functional data to reduce variant classification inequities in underrepresented populations. Genome Med 16, 143. 10.1186/s13073-024-01392-7 39627863 PMC11616159

[R16] den Dunnen J.T., Dalgleish R., Maglott D.R., Hart R.K., Greenblatt M.S., McGowan-Jordan J., Roux A.-F., Smith T., Antonarakis S.E., Taschner P.E.M., 2016. HGVS Recommendations for the Description of Sequence Variants: 2016 Update. Human Mutation 37, 564–569. 10.1002/humu.22981 26931183

[R17] DiStefano M.T., Goehringer S., Babb L., Alkuraya F.S., Amberger J., Amin M., Austin-Tse C., Balzotti M., Berg J.S., Birney E., Bocchini C., Bruford E.A., Coffey A.J., Collins H., Cunningham F., Daugherty L.C., Einhorn Y., Firth H.V., Fitzpatrick D.R., Foulger R.E., Goldstein J., Hamosh A., Hurles M.R., Leigh S.E., Leong I.U.S., Maddirevula S., Martin C.L., McDonagh E.M., Olry A., Puzriakova A., Radtke K., Ramos E.M., Rath A., Riggs E.R., Roberts A.M., Rodwell C., Snow C., Stark Z., Tahiliani J., Tweedie S., Ware J.S., Weller P., Williams E., Wright C.F., Yates T.M., Rehm H.L., 2022. The Gene Curation Coalition: A global effort to harmonize gene–disease evidence resources. Genetics in Medicine 24, 1732–1742. 10.1016/j.gim.2022.04.017 35507016 PMC7613247

[R18] Esposito D., Weile J., Shendure J., Starita L.M., Papenfuss A.T., Roth F.P., Fowler D.M., Rubin A.F., 2019. MaveDB: an open-source platform to distribute and interpret data from multiplexed assays of variant effect. Genome Biology 20, 223. 10.1186/s13059-019-1845-6 31679514 PMC6827219

[R19] Fayer S., Horton C., Dines J.N., Rubin A.F., Richardson M.E., McGoldrick K., Hernandez F., Pesaran T., Karam R., Shirts B.H., Fowler D.M., Starita L.M., 2021. Closing the gap: Systematic integration of multiplexed functional data resolves variants of uncertain significance in BRCA1, TP53, and PTEN. Am J Hum Genet 108, 2248–2258. 10.1016/j.ajhg.2021.11.001 34793697 PMC8715144

[R20] Federer L.M., 2022. Long-term availability of data associated with articles in PLOS ONE. PLoS One 17, e0272845. 10.1371/journal.pone.0272845 36001577 PMC9401135

[R21] Findlay G.M., 2021. Linking genome variants to disease: scalable approaches to test the functional impact of human mutations. Hum Mol Genet 30, R187–R197. 10.1093/hmg/ddab219 34338757 PMC8490018

[R22] Findlay G.M., Daza R.M., Martin B., Zhang M.D., Leith A.P., Gasperini M., Janizek J.D., Huang X., Starita L.M., Shendure J., 2018. Accurate classification of BRCA1 variants with saturation genome editing. Nature 562, 217–222. 10.1038/s41586-018-0461-z 30209399 PMC6181777

[R23] Fortuno C., Pesaran T., Dolinsky J., Yussuf A., McGoldrick K., Tavtigian S.V., Goldgar D., Spurdle A.B., James P.A., 2021. An updated quantitative model to classify missense variants in the TP53 gene: A novel multifactorial strategy. Hum Mutat 42, 1351–1361. 10.1002/humu.24264 34273903

[R24] Fowler D.M., Fields S., 2014. Deep mutational scanning: a new style of protein science. Nat Meth 11, 801–807. 10.1038/nmeth.3027 PMC4410700 25075907

[R25] Gebbia M., Zimmerman D., Jiang R., Nguyen M., Weile J., Li R., Gavac M., Kishore N., Sun S., Boonen R.A., Hamilton R., Dines J.N., Wahl A., Reuter J., Johnson B., Fowler D.M., Couch F.J., van Attikum H., Roth F.P., 2024. A missense variant effect map for the human tumor-suppressor protein CHK2. Am J Hum Genet 111, 2675–2692. 10.1016/j.ajhg.2024.10.013 39642869 PMC11639082

[R26] Gersing S., Cagiada M., Gebbia M., Gjesing A.P., Coté A.G., Seesankar G., Li R., Tabet D., Weile J., Stein A., Gloyn A.L., Hansen T., Roth F.P., Lindorff-Larsen K., Hartmann-Petersen R., 2023. A comprehensive map of human glucokinase variant activity. Genome Biol 24, 97. 10.1186/s13059-023-02935-8 37101203 PMC10131484

[R27] Gersing S., Schulze T.K., Cagiada M., Stein A., Roth F.P., Lindorff-Larsen K., Hartmann-Petersen R., 2024. Characterizing glucokinase variant mechanisms using a multiplexed abundance assay. Genome Biol 25, 98. 10.1186/s13059-024-03238-2 38627865 PMC11021015

[R28] Giacomelli A.O., Yang X., Lintner R.E., McFarland J.M., Duby M., Kim J., Howard T.P., Takeda D.Y., Ly S.H., Kim E., Gannon H.S., Hurhula B., Sharpe T., Goodale A., Fritchman B., Steelman S., Vazquez F., Tsherniak A., Aguirre A.J., Doench J.G., Piccioni F., Roberts C.W.M., Meyerson M., Getz G., Johannessen C.M., Root D.E., Hahn W.C., 2018. Mutational processes shape the landscape of TP53 mutations in human cancer. Nature Genetics 1. 10.1038/s41588-018-0204-y PMC6168352 30224644

[R29] Gilbert M.A., Keefer-Jacques E., Jadhav T., Antfolk D., Ming Q., Valente N., Shaw G.T.-W., Sottolano C.J., Matwijec G., Luca V.C., Loomes K.M., Rajagopalan R., Hayeck T.J., Spinner N.B., 2024. Functional characterization of 2,832 JAG1 variants supports reclassification for Alagille syndrome and improves guidance for clinical variant interpretation. Am J Hum Genet 111, 1656–1672. 10.1016/j.ajhg.2024.06.011 39043182 PMC11339624

[R30] Glazer A.M., Kroncke B.M., Matreyek K.A., Yang T., Wada Y., Shields T., Salem J.-E., Fowler D.M., Roden D.M., 2020. Deep Mutational Scan of an SCN5A Voltage Sensor. Circ Genom Precis Med 13, e002786. 10.1161/CIRCGEN.119.002786 31928070 PMC7031040

[R31] Grønbæk-Thygesen M., Voutsinos V., Johansson K.E., Schulze T.K., Cagiada M., Pedersen L., Clausen L., Nariya S., Powell R.L., Stein A., Fowler D.M., Lindorff-Larsen K., Hartmann-Petersen R., 2024. Deep mutational scanning reveals a correlation between degradation and toxicity of thousands of aspartoacylase variants. Nat Commun 15, 4026. 10.1038/s41467-024-48481-0 38740822 PMC11091098

[R32] Hart R.K., Prlić A., 2020. SeqRepo: A system for managing local collections of biological sequences. PLoS One 15, e0239883. 10.1371/journal.pone.0239883 33270643 PMC7714221

[R33] Hu C., Huang H., Na J., Lumby C., Abozaid M., Holdren M.A., Rao T.J., Karam R., Pesaran T., Weyandt J.D., Csuy C.M., Seelaus C.A., Young C.C., Fulk K., Heidari Z., Morais Lyra P.C., Couch R.E., Persons B., Polley E.C., Gnanaolivu R.D., Boddicker N.J., Monteiro A.N.A., Yadav S., Domchek S.M., Richardson M.E., Couch F.J., 2024. Functional analysis and clinical classification of 462 germline BRCA2 missense variants affecting the DNA binding domain. Am J Hum Genet 111, 584–593. 10.1016/j.ajhg.2024.02.002 38417439 PMC10940015

[R34] Consortium IGVF, 2024. Deciphering the impact of genomic variation on function. Nature 633, 47–57. 10.1038/s41586-024-07510-0 39232149 PMC11973978

[R35] Jia X., Burugula B.B., Chen V., Lemons R.M., Jayakody S., Maksutova M., Kitzman J.O., 2021. Massively parallel functional testing of MSH2 missense variants conferring Lynch syndrome risk. Am J Hum Genet 108, 163–175. 10.1016/j.ajhg.2020.12.003 33357406 PMC7820803

[R36] Jiang C., Richardson E., Farr J., Hill A.P., Ullah R., Kroncke B.M., Harrison S.M., Thomson K.L., Ingles J., Vandenberg J.I., Ng C.-A., 2022. A calibrated functional patch-clamp assay to enhance clinical variant interpretation in KCNH2-related long QT syndrome. Am J Hum Genet 109, 1199–1207. 10.1016/j.ajhg.2022.05.002 35688147 PMC9300752

[R37] Kato S., Han S.-Y., Liu W., Otsuka K., Shibata H., Kanamaru R., Ishioka C., 2003. Understanding the function–structure and function–mutation relationships of p53 tumor suppressor protein by high-resolution missense mutation analysis. PNAS 100, 8424–8429. 10.1073/pnas.1431692100 12826609 PMC166245

[R38] Kozek K.A., Glazer A.M., Ng C.-A., Blackwell D., Egly C.L., Vanags L.R., Blair M., Mitchell D., Matreyek K.A., Fowler D.M., Knollmann B.C., Vandenberg J.I., Roden D.M., Kroncke B.M., 2020. High-throughput discovery of trafficking-deficient variants in the cardiac potassium channel KV11.1. Heart Rhythm 17, 2180–2189. 10.1016/j.hrthm.2020.05.041 32522694 PMC7704534

[R39] Landrum M.J., Chitipiralla S., Kaur K., Brown G., Chen C., Hart J., Hoffman D., Jang W., Liu C., Maddipatla Z., Maiti R., Mitchell J., Rezaie T., Riley G., Song G., Yang J., Ziyabari L., Russette A., Kattman B.L., 2025. ClinVar: updates to support classifications of both germline and somatic variants. Nucleic Acids Res 53, D1313–D1321. 10.1093/nar/gkae1090 39578691 PMC11701624

[R40] Lee K., Abul-Husn N.S., Amendola L.M., Brothers K.B., Chung W.K., Gollob M.H., Gordon A.S., Harrison S.M., Hershberger R.E., Li M., Ondrasik D., Richards C.S., Stergachis A., Stewart D.R., Martin C.L., Miller D.T., ACMG Secondary Findings Working Group. Electronic address: documents@acmg.net, 2025. ACMG SF v3.3 list for reporting of secondary findings in clinical exome and genome sequencing: A policy statement of the American College of Medical Genetics and Genomics (ACMG). Genet Med 27, 101454. 10.1016/j.gim.2025.101454 40568962 PMC12318660

[R41] Lemaire B., Bauer F., Chaves Rodriguez E., Ghesquiere J., Radziejwoski A., Roth A., Boyer M., 2025. Web references are not eternal: time-trend and qualitative impact of the loss of access to online resources cited in peer-reviewed medical journals. Curr Med Res Opin 41, 543–548. 10.1080/03007995.2025.2475091 40040548

[R42] Li C., Wilborn J., Pittman S., Daw J., Alonso-Pérez J., Díaz-Manera J., Weihl C.C., Haller G., 2023. Comprehensive functional characterization of SGCB coding variants predicts pathogenicity in limb-girdle muscular dystrophy type R4/2E. J Clin Invest 133, e168156. 10.1172/JCI168156 37317968 PMC10266784

[R43] Lo R.S., Cromie G.A., Tang M., Teng K., Owens K., Sirr A., Kutz J.N., Morizono H., Caldovic L., Ah Mew N., Gropman A., Dudley A.M., 2023. The functional impact of 1,570 individual amino acid substitutions in human OTC. Am J Hum Genet 110, 863–879. 10.1016/j.ajhg.2023.03.019 37146589 PMC10183466

[R44] Ma J.G., O’Neill M.J., Richardson E., Thomson K.L., Ingles J., Muhammad A., Solus J.F., Davogustto G., Anderson K.C., Shoemaker M.B., Stergachis A.B., Floyd B.J., Dunn K., Parikh V.N., Chubb H., Perrin M.J., Roden D.M., Vandenberg J.I., Ng C.-A., Glazer A.M., 2024. Multisite Validation of a Functional Assay to Adjudicate SCN5A Brugada Syndrome-Associated Variants. Circ Genom Precis Med 17, e004569. 10.1161/CIRCGEN.124.004569 38953211 PMC11335442

[R45] Ma K., Huang S., Ng K.K., Lake N.J., Joseph S., Xu J., Lek A., Ge L., Woodman K.G., Koczwara K.E., Cohen J., Ho V., O’Connor C.L., Brindley M.A., Campbell K.P., Lek M., 2024. Saturation mutagenesis-reinforced functional assays for disease-related genes. Cell 187, 6707–6724.e22. 10.1016/j.cell.2024.08.047 39326416 PMC11568926

[R46] Martin B.E., Sands T., Bier L., Bergner A., Boehme A.K., Lippa N., 2024. Comparing the frequency of variants of uncertain significance (VUS) between ancestry groups in a paediatric epilepsy cohort. J Med Genet 61, 645–651. 10.1136/jmg-2023-109450 38453479

[R47] Matreyek K.A., Starita L.M., Stephany J.J., Martin B., Chiasson M.A., Gray V.E., Kircher M., Khechaduri A., Dines J.N., Hause R.J., Bhatia S., Evans W.E., Relling M.V., Yang W., Shendure J., Fowler D.M., 2018. Multiplex assessment of protein variant abundance by massively parallel sequencing. Nature Genetics 50, 874–882. 10.1038/s41588-018-0122-z 29785012 PMC5980760

[R48] McDonnell A.F., Plech M., Livesey B.J., Gerasimavicius L., Owen L.J., Hall H.N., FitzPatrick D.R., Marsh J.A., Kudla G., 2024. Deep mutational scanning quantifies DNA binding and predicts clinical outcomes of PAX6 variants. Mol Syst Biol 20, 825–844. 10.1038/s44320-024-00043-8 38849565 PMC11219921

[R49] McEwen A.E., Tejura M., Fayer S., Starita L.M., Fowler D.M., 2025. Multiplexed assays of variant effect for clinical variant interpretation. Nat Rev Genet. 10.1038/s41576-025-00870-x 40691352

[R50] Meitlis I., Allenspach E.J., Bauman B.M., Phan I.Q., Dabbah G., Schmitt E.G., Camp N.D., Torgerson T.R., Nickerson D.A., Bamshad M.J., Hagin D., Luthers C.R., Stinson J.R., Gray J., Lundgren I., Church J.A., Butte M.J., Jordan M.B., Aceves S.S., Schwartz D.M., Milner J.D., Schuval S., Skoda-Smith S., Cooper M.A., Starita L.M., Rawlings D.J., Snow A.L., James R.G., 2020. Multiplexed Functional Assessment of Genetic Variants in CARD11. Am J Hum Genet 107, 1029–1043. 10.1016/j.ajhg.2020.10.015 33202260 PMC7820631

[R51] Mighell T.L., Evans-Dutson S., O’Roak B.J., 2018. A Saturation Mutagenesis Approach to Understanding PTEN Lipid Phosphatase Activity and Genotype-Phenotype Relationships. The American Journal of Human Genetics 102, 943–955. 10.1016/j.ajhg.2018.03.018 29706350 PMC5986715

[R52] Mighton C., Smith A.C., Mayers J., Tomaszewski R., Taylor S., Hume S., Agatep R., Spriggs E., Feilotter H.E., Semenuk L., Wong H., Lazo de la Vega L., Marshall C.R., Axford M.M., Silver T., Charames G.S., Di Gioacchino V., Watkins N., Foulkes W.D., Clavier M., Hamel N., Chong G., Lamont R.E., Parboosingh J., Karsan A., Bosdet I., Young S.S., Tucker, Akbari M.R., Speevak M.D., Vaags A.K., Lebo M.S., Lerner-Ellis J., Canadian Open Genetics Repository Working Group, 2022. Data sharing to improve concordance in variant interpretation across laboratories: results from the Canadian Open Genetics Repository. J Med Genet 59, 571–578. 10.1136/jmedgenet-2021-107738 33875564 PMC8523590

[R53] Muhammad A., Calandranis M.E., Li B., Yang T., Blackwell D.J., Harvey M.L., Smith J.E., Daniel Z.A., Chew A.E., Capra J.A., Matreyek K.A., Fowler D.M., Roden D.M., Glazer A.M., 2024. High-throughput functional mapping of variants in an arrhythmia gene, KCNE1, reveals novel biology. Genome Med 16, 73. 10.1186/s13073-024-01340-5 38816749 PMC11138074

[R54] Ndugga-Kabuye M.K., Issaka R.B., 2019. Inequities in multi-gene hereditary cancer testing: lower diagnostic yield and higher VUS rate in individuals who identify as Hispanic, African or Asian and Pacific Islander as compared to European. Fam Cancer 18, 465–469. 10.1007/s10689-019-00144-6 31531760 PMC6785397

[R55] Olvera-León R., Zhang F., Offord V., Zhao Y., Tan H.K., Gupta P., Pal T., Robles-Espinoza C.D., Arriaga-González F.G., Matsuyama L.S.A.S., Delage E., Dicks E., Ezquina S., Rowlands C.F., Turnbull C., Pharoah P., Perry J.R.B., Jasin M., Waters A.J., Adams D.J., 2024. High-resolution functional mapping of RAD51C by saturation genome editing. Cell 187, 5719–5734.e19. 10.1016/j.cell.2024.08.039 39299233 PMC12147941

[R56] O’Neill M.J., Ng C.-A., Aizawa T., Sala L., Bains S., Winbo A., Ullah R., Shen Q., Tan C.-Y., Kozek K., Vanags L.R., Mitchell D.W., Shen A., Wada Y., Kashiwa A., Crotti L., Dagradi F., Musu G., Spazzolini C., Neves R., Bos J.M., Giudicessi J.R., Bledsoe X., Gamazon E.R., Lancaster M.C., Glazer A.M., Knollmann B.C., Roden D.M., Weile J., Roth F., Salem J.-E., Earle N., Stiles R., Agee T., Johnson C.N., Horie M., Skinner J.R., Ackerman M.J., Schwartz P.J., Ohno S., Vandenberg J.I., Kroncke B.M., 2024. Multiplexed Assays of Variant Effect and Automated Patch Clamping Improve KCNH2-LQTS Variant Classification and Cardiac Event Risk Stratification. Circulation 150, 1869–1881. 10.1161/CIRCULATIONAHA.124.069828 39315434 PMC11611689

[R57] Park M.S., Kumar R.D., Ovadiuc C., Folta A., McEwen A.E., Snyder A., Villani R.M., Spurdle A.B., Fowler D.M., Rubin A.F., Shirts B.H., Starita L.M., Stergachis A.B., 2025. Insights on improving accessibility and usability of functional data to unlock their potential for variant interpretation. Am J Hum Genet 112, 1468–1478. 10.1016/j.ajhg.2025.04.009 40480199 PMC12256793

[R58] Pawliczek P., Patel R.Y., Ashmore L.R., Jackson A.R., Bizon C., Nelson T., Powell B., Freimuth R.R., Strande N., Shah N., Paithankar S., Wright M.W., Dwight S., Zhen J., Landrum M., McGarvey P., Babb L., Plon S.E., Milosavljevic A., Clinical Genome (ClinGen) Resource, 2018. ClinGen Allele Registry links information about genetic variants. Hum Mutat 39, 1690–1701. 10.1002/humu.23637 30311374 PMC6519371

[R59] Popp N.A., Powell R.L., Wheelock M.K., Holmes K.J., Zapp B.D., Sheldon K.M., Fletcher S.N., Wu X., Fayer S., Rubin A.F., Lannert K.W., Chang A.T., Sheehan J.P., Johnsen J.M., Fowler D.M., 2025. Multiplex and multimodal mapping of variant effects in secreted proteins via MultiSTEP. Nat Struct Mol Biol. 10.1038/s41594-025-01582-w PMC12373428 40514537

[R60] Radford E.J., Tan H.-K., Andersson M.H.L., Stephenson J.D., Gardner E.J., Ironfield H., Waters A.J., Gitterman D., Lindsay S., Abascal F., Martincorena I., Kolesnik-Taylor A., Ng-Cordell E., Firth H.V., Baker K., Perry J.R.B., Adams D.J., Gerety S.S., Hurles M.E., 2023. Saturation genome editing of DDX3X clarifies pathogenicity of germline and somatic variation. Nat Commun 14, 7702. 10.1038/s41467-023-43041-4 38057330 PMC10700591

[R61] Rehm H.L., Alaimo J.T., Aradhya S., Bayrak-Toydemir P., Best H., Brandon R., Buchan J.G., Chao E.C., Chen E., Clifford J., Cohen A.S.A., Conlin L.K., Das S., Davis K.W., Del Gaudio D., Del Viso F., DiVincenzo C., Eisenberg M., Guidugli L., Hammer M.B., Harrison S.M., Hatchell K.E., Dyer L.H., Hoang L.U., Holt J.M., Jobanputra V., Karbassi I.D., Kearney H.M., Kelly M.A., Kelly J.M., Kluge M.L., Komala T., Kruszka P., Lau L., Lebo M.S., Marshall C.R., McKnight D., McWalter K., Meng Y., Nagan N., Neckelmann C.S., Neerman N., Niu Z., Paolillo V.K., Paolucci S.A., Perry D., Pesaran T., Radtke K., Rasmussen K.J., Retterer K., Saunders C.J., Spiteri E., Stanley C., Szuto A., Taft R.J., Thiffault I., Thomas B.C., Thomas-Wilson A., Thorpe E., Tidwell T.J., Towne M.C., Zouk H., Medical Genome Initiative Steering Committee, 2023. The landscape of reported VUS in multi-gene panel and genomic testing: Time for a change. Genet Med 25, 100947. 10.1016/j.gim.2023.100947 37534744 PMC10825061

[R62] Rehm H.L., Page A.J.H., Smith L., Adams J.B., Alterovitz G., Babb L.J., Barkley M.P., Baudis M., Beauvais M.J.S., Beck T., Beckmann J.S., Beltran S., Bernick D., Bernier A., Bonfield J.K., Boughtwood T.F., Bourque G., Bowers S.R., Brookes A.J., Brudno M., Brush M.H., Bujold D., Burdett T., Buske O.J., Cabili M.N., Cameron D.L., Carroll R.J., Casas-Silva E., Chakravarty D., Chaudhari B.P., Chen S.H., Cherry J.M., Chung J., Cline M., Clissold H.L., Cook-Deegan R.M., Courtot M., Cunningham F., Cupak M., Davies R.M., Denisko D., Doerr M.J., Dolman L.I., Dove E.S., Dursi L.J., Dyke S.O.M., Eddy J.A., Eilbeck K., Ellrott K.P., Fairley S., Fakhro K.A., Firth H.V., Fitzsimons M.S., Fiume M., Flicek P., Fore I.M., Freeberg M.A., Freimuth R.R., Fromont L.A., Fuerth J., Gaff C.L., Gan W., Ghanaim E.M., Glazer D., Green R.C., Griffith M., Griffith O.L., Grossman R.L., Groza T., Auvil J.M.G., Guigó R., Gupta D., Haendel M.A., Hamosh A., Hansen D.P., Hart R.K., Hartley D.M., Haussler D., Hendricks-Sturrup R.M., Ho C.W.L., Hobb A.E., Hoffman M.M., Hofmann O.M., Holub P., Hsu J.S., Hubaux J.-P., Hunt S.E., Husami A., Jacobsen J.O., Jamuar S.S., Janes E.L., Jeanson F., Jené A., Johns A.L., Joly Y., Jones S.J.M., Kanitz A., Kato K., Keane T.M., Kekesi-Lafrance K., Kelleher J., Kerry G., Khor S.-S., Knoppers B.M., Konopko M.A., Kosaki K., Kuba M., Lawson J., Leinonen R., Li S., Lin M.F., Linden M., Liu X., Udara Liyanage I., Lopez J., Lucassen A.M., Lukowski M., Mann A.L., Marshall J., Mattioni M., Metke-Jimenez A., Middleton A., Milne R.J., Molnár-Gábor F., Mulder N., Munoz-Torres M.C., Nag R., Nakagawa H., Nasir J., Navarro A., Nelson T.H., Niewielska A., Nisselle A., Niu J., Nyrönen T.H., O’Connor B.D., Oesterle S., Ogishima S., Wang V.O., Paglione L.A.D., Palumbo E., Parkinson H.E., Philippakis A.A., Pizarro A.D., Prlic A., Rambla J., Rendon A., Rider R.A., Robinson P.N., Rodarmer K.W., Rodriguez L.L., Rubin A.F., Rueda M., Rushton G.A., Ryan R.S., Saunders G.I., Schuilenburg H., Schwede T., Scollen S., Senf A., Sheffield N.C., Skantharajah N., Smith A.V., Sofia H.J., Spalding D., Spurdle A.B., Stark Z., Stein L.D., Suematsu M., Tan P., Tedds J.A., Thomson A.A., Thorogood A., Tickle T.L., Tokunaga K., Törnroos J., Torrents D., Upchurch S., Valencia A., Guimera R.V., Vamathevan J., Varma S., Vears D.F., Viner C., Voisin C., Wagner A.H., Wallace S.E., Walsh B.P., Williams M.S., Winkler E.C., Wold B.J., Wood G.M., Woolley J.P., Yamasaki C., Yates A.D., Yung C.K., Zass L.J., Zaytseva K., Zhang J., Goodhand P., North K., Birney E., 2021. GA4GH: International policies and standards for data sharing across genomic research and healthcare. Cell Genom 1, 100029. 10.1016/j.xgen.2021.100029 35072136 PMC8774288

[R63] Richards S., Aziz N., Bale S., Bick D., Das S., Gastier-Foster J., Grody W.W., Hegde M., Lyon E., Spector E., Voelkerding K., Rehm H.L., ACMG Laboratory Quality Assurance Committee, 2015. Standards and guidelines for the interpretation of sequence variants: a joint consensus recommendation of the American College of Medical Genetics and Genomics and the Association for Molecular Pathology. Genet Med 17, 405–424. 10.1038/gim.2015.30 25741868 PMC4544753

[R64] Rubin A.F., Stone J., Bianchi A.H., Capodanno B.J., Da E.Y., Dias M., Esposito D., Frazer J., Fu Y., Grindstaff S.B., Harrington M.R., Li I., McEwen A.E., Min J.K., Moore N., Moscatelli O.G., Ong J., Polunina P.V., Rollins J.E., Rollins N.J., Snyder A.E., Tam A., Wakefield M.J., Ye S.S., Starita L.M., Bryant V.L., Marks D.S., Fowler D.M., 2025. MaveDB 2024: a curated community database with over seven million variant effects from multiplexed functional assays. Genome Biol 26, 13. 10.1186/s13059-025-03476-y 39838450 PMC11753097

[R65] Sahu S., Galloux M., Southon E., Caylor D., Sullivan T., Arnaudi M., Zanti M., Geh J., Chari R., Michailidou K., Papaleo E., Sharan S.K., 2025. Saturation genome editing-based clinical classification of BRCA2 variants. Nature 638, 538–545. 10.1038/s41586-024-08349-1 39779848

[R66] Sahu S., Sullivan T.L., Mitrophanov A.Y., Galloux M., Nousome D., Southon E., Caylor D., Mishra A.P., Evans C.N., Clapp M.E., Burkett S., Malys T., Chari R., Biswas K., Sharan S.K., 2023. Saturation genome editing of 11 codons and exon 13 of BRCA2 coupled with chemotherapeutic drug response accurately determines pathogenicity of variants. PLoS Genet 19, e1010940. 10.1371/journal.pgen.1010940 37713444 PMC10529611

[R67] Shepherdson J.L., Granas D.M., Li J., Shariff Z., Plassmeyer S.P., Holehouse A.S., White M.A., Cohen B.A., 2024. Mutational scanning of CRX classifies clinical variants and reveals biochemical properties of the transcriptional effector domain. Genome Res 34, 1540–1552. 10.1101/gr.279415.124 39322280 PMC11529990

[R68] Starita L.M., Ahituv N., Dunham M.J., Kitzman J.O., Roth F.P., Seelig G., Shendure J., Fowler D.M., 2017. Variant Interpretation: Functional Assays to the Rescue. The American Journal of Human Genetics 101, 315–325. 10.1016/j.ajhg.2017.07.014 28886340 PMC5590843

[R69] Sun S., Weile J., Verby M., Wu Y., Wang Y., Cote A.G., Fotiadou I., Kitaygorodsky J., Vidal M., Rine J., Ješina P., Kožich V., Roth F.P., 2020. A proactive genotype-to-patient-phenotype map for cystathionine beta-synthase. Genome Medicine 12, 13. 10.1186/s13073-020-0711-1 32000841 PMC6993387

[R70] Sung A.Y., Guerra R.M., Steenberge L.H., Alston C.L., Murayama K., Okazaki Y., Shimura M., Prokisch H., Ghezzi D., Torraco A., Carrozzo R., Rötig A., Taylor R.W., Keck J.L., Pagliarini D.J., 2024. Systematic analysis of NDUFAF6 in complex I assembly and mitochondrial disease. Nat Metab 6, 1128–1142. 10.1038/s42255-024-01039-2 38720117 PMC11395703

[R71] Tabet D., Parikh V., Mali P., Roth F.P., Claussnitzer M., 2022. Scalable Functional Assays for the Interpretation of Human Genetic Variation. Annu Rev Genet 56, 441–465. 10.1146/annurev-genet-072920-032107 36055970

[R72] Tavtigian S.V., Greenblatt M.S., Harrison S.M., Nussbaum R.L., Prabhu S.A., Boucher K.M., Biesecker L.G., ClinGen Sequence Variant Interpretation Working Group (ClinGen SVI), 2018. Modeling the ACMG/AMP variant classification guidelines as a Bayesian classification framework. Genet Med 20, 1054–1060. 10.1038/gim.2017.210 29300386 PMC6336098

[R73] van Loggerenberg W., Sowlati-Hashjin S., Weile J., Hamilton R., Chawla A., Sheykhkarimli D., Gebbia M., Kishore N., Frésard L., Mustajoki S., Pischik E., Di Pierro E., Barbaro M., Floderus Y., Schmitt C., Gouya L., Colavin A., Nussbaum R., Friesema E.C.H., Kauppinen R., To-Figueras J., Aarsand A.K., Desnick R.J., Garton M., Roth F.P., 2023. Systematically testing human HMBS missense variants to reveal mechanism and pathogenic variation. Am J Hum Genet 110, 1769–1786. 10.1016/j.ajhg.2023.08.012 37729906 PMC10577081

[R74] Villani R.M., Terrill B., Tudini E., McKenzie M.E., Cliffe C.C., Hahn C.N., Lundie B., Mattiske T., Matotek E., McEwen A.E., Nickerson S.L., Breen J., Fowler D.M., Christodoulou J., Starita L., Rubin A.F., Spurdle A.B., 2025. Consultation informs strategies for improving the use of functional evidence in variant classification. Am J Hum Genet 112, 1489–1495. 10.1016/j.ajhg.2025.05.003 40480201 PMC12256917

[R75] Wagner A.H., Babb L., Alterovitz G., Baudis M., Brush M., Cameron D.L., Cline M., Griffith M., Griffith O.L., Hunt S.E., Kreda D., Lee J.M., Li S., Lopez J., Moyer E., Nelson T., Patel R.Y., Riehle K., Robinson P.N., Rynearson S., Schuilenburg H., Tsukanov K., Walsh B., Konopko M., Rehm H.L., Yates A.D., Freimuth R.R., Hart R.K., 2021. The GA4GH Variation Representation Specification: A computational framework for variation representation and federated identification. Cell Genom 1, 100027. 10.1016/j.xgen.2021.100027 35311178 PMC8929418

[R76] Wan A., Place E., Pierce E.A., Comander J., 2019. Characterizing variants of unknown significance in rhodopsin: A functional genomics approach. Hum Mutat 40, 1127–1144. 10.1002/humu.23762 30977563 PMC7027811

[R77] Waters A.J., Brendler-Spaeth T., Smith D., Offord V., Tan H.K., Zhao Y., Obolenski S., Nielsen M., van Doorn R., Murphy J.-E., Gupta P., Rowlands C.F., Hanson H., Delage E., Thomas M., Radford E.J., Gerety S.S., Turnbull C., Perry J.R.B., Hurles M.E., Adams D.J., 2024. Saturation genome editing of BAP1 functionally classifies somatic and germline variants. Nat Genet 56, 1434–1445. 10.1038/s41588-024-01799-3 38969833 PMC11250367

[R78] Weile J., Roth F.P., 2018. Multiplexed assays of variant effects contribute to a growing genotype-phenotype atlas. Hum Genet 137, 665–678. 10.1007/s00439-018-1916-x 30073413 PMC6153521

[R79] Weile J., Sun S., Cote A.G., Knapp J., Verby M., Mellor J.C., Wu Y., Pons C., Wong C., Lieshout N. van, Yang F., Tasan M., Tan G., Yang S., Fowler D.M., Nussbaum R., Bloom J.D., Vidal M., Hill D.E., Aloy P., Roth F.P., 2017. A framework for exhaustively mapping functional missense variants. Molecular Systems Biology 13, 957. 10.15252/msb.20177908 29269382 PMC5740498

[R80] Zeiberg D., Tejura M., McEwen A.E., Fayer S., Pejaver V., Rubin A.F., Starita L.M., Fowler D.M., O’Donnell-Luria A., Radivojac P., 2025. Gene-based calibration of high-throughput functional assays for clinical variant classification. bioRxiv 2025.04.29.651326. 10.1101/2025.04.29.651326

[R81] Zheng H., Yan X., Li G., Lin H., Deng S., Zhuang W., Yao F., Lu Y., Xia X., Yuan H., Jin L., Yan Z., 2022. Proactive functional classification of all possible missense single-nucleotide variants in KCNQ4. Genome Res 32, 1573–1584. 10.1101/gr.276562.122 35760561 PMC9435748

