# Supplementary material for: MaveMD: A functional data resource for genomic medicine
Source: medRxiv. 2025 Nov 19:2025.11.15.25336228. Preprint. [Version 1] doi: 10.1101/2025.11.15.25336228 (PMC12668102; doi:10.1101/2025.11.15.25336228)
Supplement: 1 [file NIHPP2025.11.15.25336228V1-supplement-1.pdf]

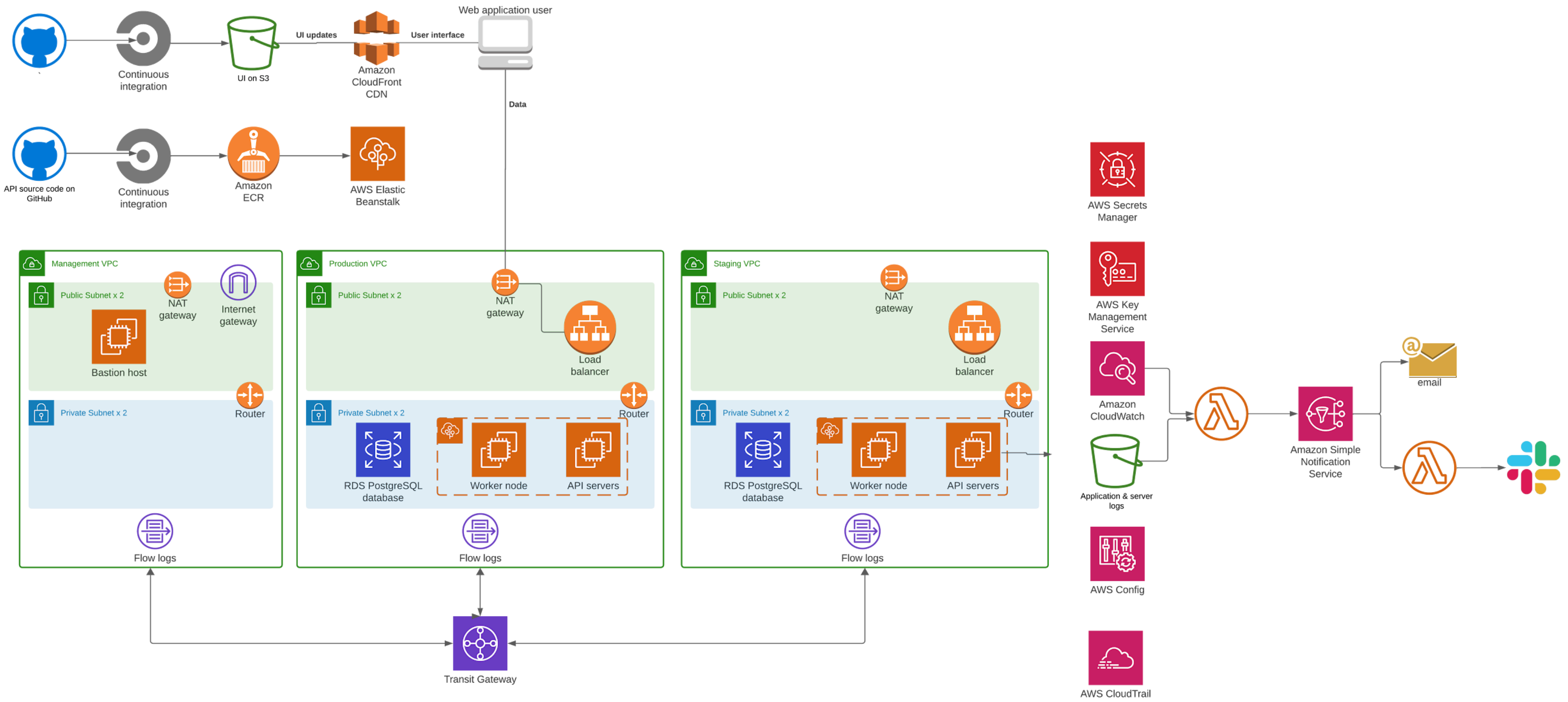

Supplemental Figure S1: Amazon Web Services (AWS) architecture diagram. MaveDB and MaveMD are hosted in a cloud computing environment provided by AWS. This schematic shows the various processes and containers involved in running, updating, and monitoring MaveDB and MaveMD.

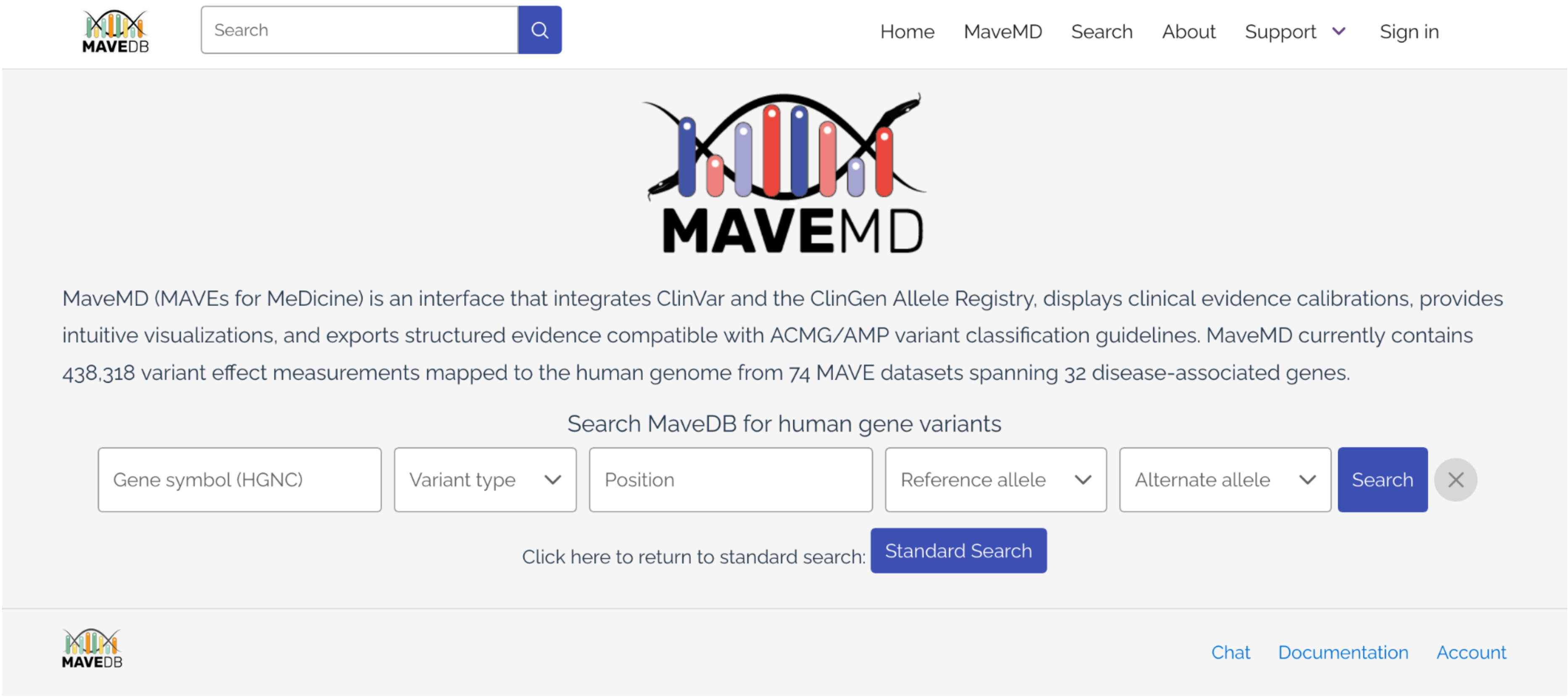

Supplemental Figure S2: Variant fuzzy search form. This screenshot shows the search interface for the variant fuzzy search implemented in MaveMD. Variant type, reference allele, and alternate allele are dropdown selections, and gene symbol and position are free text. Input to this form is validated and passed to the ClinGen Allele Registry via API to resolve variant search queries.

medRxiv preprint doi: https://doi.org/10.1101/2025.11.15.25336228; this version posted November 19, 2025. The copyright holder for this preprint (which was not certified by peer review) is the author/funder, who has granted medRxiv a license to display the preprint in perpetuity.
It is made available under a CC-BY 4.0 International license .

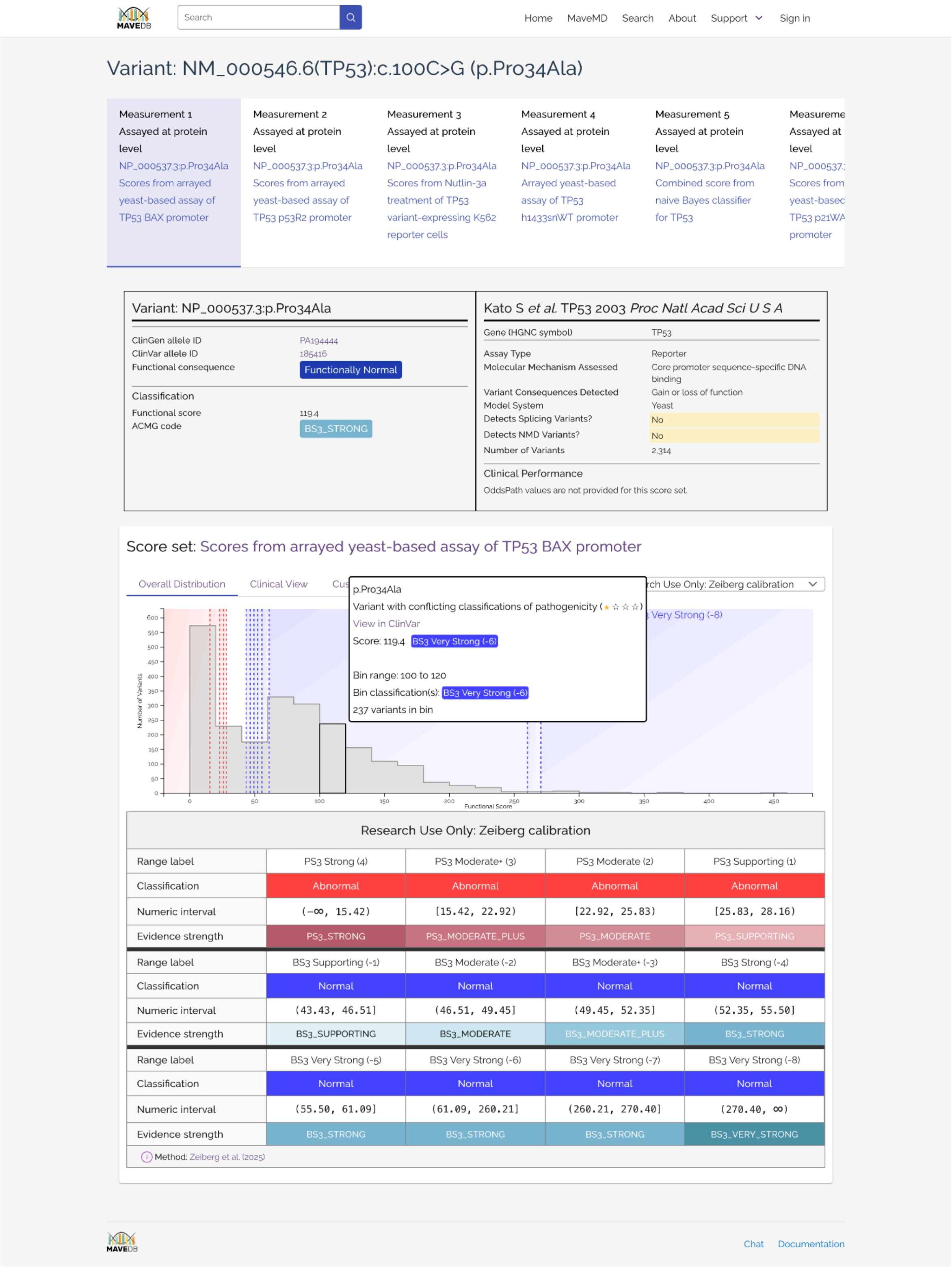

Supplemental Figure S3: Variant search results page. This screenshot shows the results page for the variant search implemented in MaveMD. Available measurements (assay results) are available at the top of the page and the user can select which one should be used for visualization. The variant is shown in HGVS format, along with alternate identifiers. The variant's position in the assay score histogram is also highlighted.

medRxiv preprint doi: https://doi.org/10.1101/2025.11.15.25336228; this version posted November 19, 2025. The copyright holder for this preprint (which was not certified by peer review) is the author/funder, who has granted medRxiv a license to display the preprint in perpetuity.
It is made available under a CC-BY 4.0 International license .

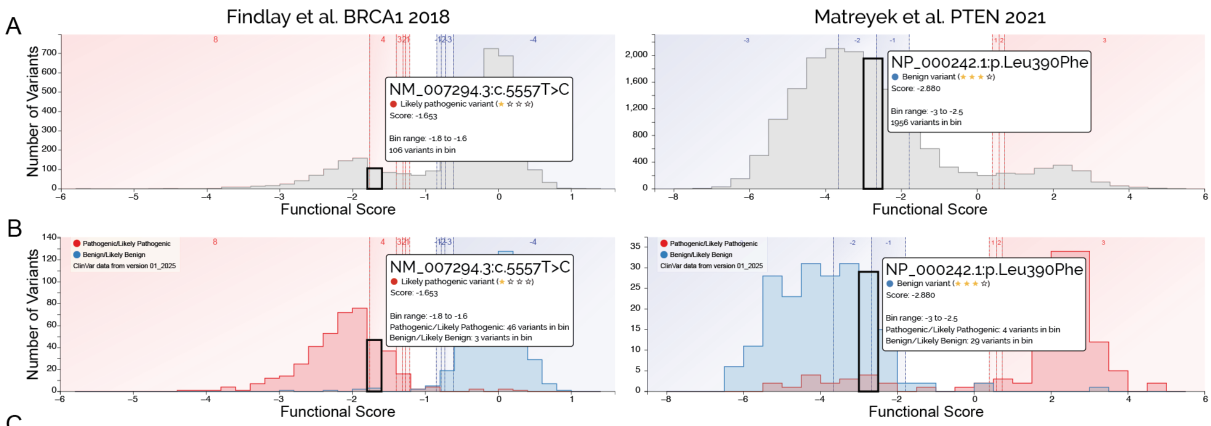

C

Research Use Only: Zeiberg calibration

8	4	3	2	1
PS3_VERY_STRONG	PS3_STRONG	PS3_MODERATE+	PS3_MODERATE	PS3_SUPPORTING
(-∞, -1.76]	(-1.76, -1.40]	(-1.40, -1.31]	(-1.31, -1.27]	(-1.27, -1.21]

-1	-2	-3	-4
BS3_SUPPORTING	BS3_MODERATE	BS3_MODERATE+	BS3_STRONG
[-0.848, -0.783)	[-0.783, -0.730)	[-0.730, -0.618)	[-0.618, ∞)

Research Use Only: Zeiberg calibration

-3	-2	-1	1	2
BS3_MODERATE+	BS3_MODERATE	BS3_SUPPORTING	PS3_SUPPORTING	PS3_MODERATE
(-∞, -1.76]	(-1.76, -1.40]	(-1.40, -1.31]	(-1.31, -1.27]	(-1.27, -1.21]

3
PS3_MODERATE+
[-0.848, -0.783)

Supplemental Figure S4: MaveMD support for alternative calibration methods. A) Interactive histograms show the full distribution of variant scores in an assay and the relative position of a selected variant. Vertical bars also denote evidence thresholds. B) Another interactive histogram displays the distribution of variant scores for variants from ClinVar. C) Clinical calibration details and the associated evidence codes and score ranges are shown in a table below the histogram. The histogram shows the calibrations based on the method described by (Zeiberg et al., 2025).
